# Supplementary material for: An Innovative Approach: The Usage of N-Acetylcysteine in the Therapy of Pneumonia in Neonatal Calves
Source: Animals (Basel). 2024 Oct 3;14(19):2852. doi: 10.3390/ani14192852 (PMC11475344; doi:10.3390/ani14192852)
Supplement: Supplementary file 1 [file animals-14-02852-s001.zip › animals-3164843-supplementary.pdf]

Supplementary Table S1. Calves clinical scoring of both N acetyl cysteine (NAC) groups before treatment.

		n	Mode	Median	Coefficient of variation	IQR	Minimum	Maximum
**Nasal discharge**	Control	20	2.000	2.000	0.365	1.000	1.000	3.000
	NAC	20	2.000	2.000	0.374	1.000	1.000	3.000
**Ocular discharge**	Control	20	1.000	1.000	0.280	0.000	1.000	2.000
	NAC	20	1.000	1.000	0.319	0.000	1.000	2.000
**Cough**	Control	20	2.000	2.000	0.109	0.000	2.000	3.000
	NAC	20	2.000	2.000	0.109	0.000	2.000	3.000
**Ear position**	Control	20	1.000	1.000	0.415	0.000	0.000	2.000
	NAC	20	1.000	1.000	0.497	0.000	0.000	2.000
**Temperature score**	Control	20	1.000	1.500	0.452	1.000	1.000	3.000
	NAC	20	1.000	1.000	0.490	1.000	1.000	3.000
**Total score**	Control	20	6.000	7.000	0.210	2.000	5.000	11.000
	NAC	20	6.000	7.000	0.209	2.000	5.000	11.000

Supplementary Table S2. Comparison of clinical parameters among two groups before treatment.

	W	p
**Nasal discharge**	224.50	0.47
**Ocular discharge**	190.00	0.65
**Cough**	200.00	1.00
**Ear position**	209.50	0.72
**Temperature score**	217.00	0.62
**Total score**	219.50	0.60
**Weight**	212.00	0.75
**Temperature (°C)**	224.50	0.52
**Mann-Whitney U test.**		

Supplementary Table S3: There is a statistically significant difference in time to resolution period between the N-acetylcysteine supplemented group and control group of calves diseased from pneumonia.

	W	p
**Time to resolution**	**352.00**	**<0.001**
**Mann-Whitney U test.**		

Supplementary Table S4. Linear regression summary for dependent variable: Time to resolution.
The beginning of linear regression model.

N=40	R= .92414373 R^2= .85404163 Adjusted R^2= .81637496 F(8,31)=22.674 p					
	b*	Std.Err. of b*	b	Std.Err. of b*	t (31)	p-value
Intercept			-1248.97	328.73	-3.80	0.00
Group	-0.56	0.07	-27.63	3.48	-7.95	0.00
Ocular discharge	0.01	0.09	1.11	6.83	0.16	0.87
Cough	0.04	0.08	5.05	9.72	0.52	0.61
Ear position	-0.01	0.11	-0.75	6.44	-0.12	0.91
Temp score	-0.13	0.21	-4.27	6.97	-0.61	0.54
Total score	0.16	0.23	2.62	3.73	0.70	0.49
Weight	0.02	0.08	0.15	0.48	0.31	0.76
Temp (C)	0.66	0.17	31.93	8.19	3.90	0.00
